# Supplementary material for: Identification of photoperiod-induced specific miRNAs in the adrenal glands of Sunite sheep (Ovis aries)
Source: Front Vet Sci. 2022 Jul 22;9:888207. doi: 10.3389/fvets.2022.888207 (PMC9354845; doi:10.3389/fvets.2022.888207)
Supplement: Supplementary file 1 [file Data_Sheet_1.ZIP › Supplementary file/miRDeep2 file.zip!/miRDeep2 file/Know_Structure/LP42A2/oar-let-7i.pdf]

miRBase precursor : oar-let-7i
Total read count : 351785
oar-let-7i read count : 351582
remaining reads : 203

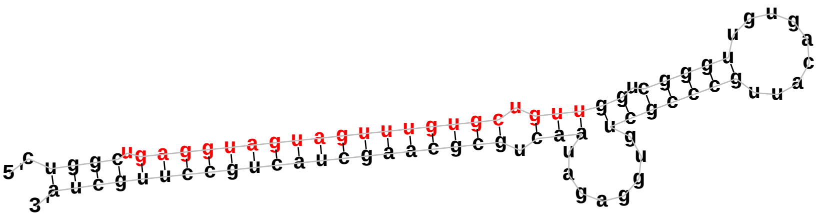

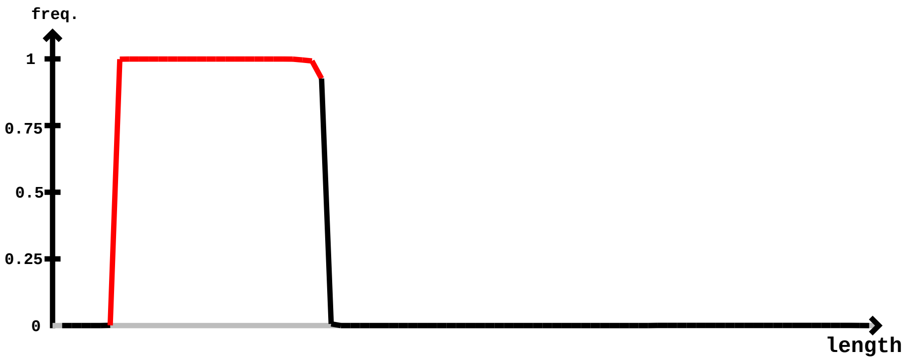


```
       oar-let-7i
5'- cuggcugagguaguaguuugugcuguuggucggguugugacauugcccgcuguggagauaacugcgcaagcuacugccuugcua   -3'    exp
    .((((.(((((((((((((((((.(((((.(((((.........)))))))........))).)))))))))))))))))))))   reads      mm         sample
    ....cugagguaguaguuugugcu............................................................   4          0          seq
    ....cugagguaguaguuugugcug...........................................................   1          0          seq
    ....cugagguaguaguuugugcugu..........................................................   25         0          seq
    ....cugagguaguaguuugugcuguu.........................................................   292        0          seq
    ....cugagguaguaguuugugcuguug........................................................   3          0          seq
    .....ugagguaguaguuugug..............................................................   13         0          seq
    .....ugagguaguaguuugugc.............................................................   74         0          seq
    .....ugagguaguaguuugugG.............................................................   2          1          seq
    .....ugagguaguaguuugugA.............................................................   13         1          seq
    .....ugagguaguaguuugugcu............................................................   1147       0          seq
    .....ugagguaguaguuugugcug...........................................................   1291       0          seq
    .....ugagguaguaguuugugcugu..........................................................   23188      0          seq
    .....ugagguaguaguuugugcuguu.........................................................   323140     0          seq
    .....ugagguaguaguuugugcuguug........................................................   2193       0          seq
    .....ugagguaguaguuugugcuguugg.......................................................   8          0          seq
    ......gagguaguaguuugugcugu..........................................................   10         0          seq
    ......gagguaguaguuugugcuguu.........................................................   173        0          seq
    ......gagguaguaguuugugcuguug........................................................   1          0          seq
    .......agguaguaguuugugcuguu.........................................................   2          0          seq
    ........gguaguaguuugugcuguu.........................................................   1          0          seq
    .........guaguaguuugugcuguu.........................................................   1          0          seq
    ...........................................ugcccgcuguggagauaacugcg..................   1          0          seq
    .............................................................cugcgcaagcuacugccuug...   5          0          seq
    .............................................................cugcgcaagcuacugccuugc..   83         0          seq
    .............................................................cugcgcaagcuacugccuugcu.   112        0          seq
    .............................................................cugcgcaagcuacugccuugcua   2          0          seq
```
